# Editorial

**Published:** 1864-12

**Authors:** 


### Editorial.

### REMINISCENCE—VALEDICTORY.

In changing any of the relations of life, some responsibility must be assumed, and, with all but the most callous, there will be more or less of mental disquiet. The cheek of the joyous bride, in that hour when love's young dream is realized, and hope culminates in possession, is bedewed with pearly drops, distilled

> "In the spirit's pure, deep fountains,
> Whence the holy passions well."

And the man who is taken from retirement to a position of notoriety, who is called to the assumption of duties which make him directly amenable to society—duties which invite the criticisms of his compeers—a modest man, he shrinks—a bashful man, he suffers, and even a presumptous man, he is not exactly at ease.

> Not that the duty's heavy,
> But simply that its new.

Nearly nine years ago I became editorially connected with The Dental Register of the West. The position implied new duties, and new responsibilities. How these have been met, and those discharged, the readers of the "Register" know. They were not lightly assumed. The whole subject was carefully considered before the final step was taken. Much labor and little reward were expected. And there has been no disappointment. That the labor has not been light, is attested by the hundreds of pages from a single pen. "What I have written I have written." Not here and now is the character of these pages to be discussed. But it is not easy work for any man to do his best. Of pecuniary reward for this labor, it is enough to say, "blessed are they that expect nothing; for they can afford to be disappointed."

Taking up the editorial pen was the occasion of much anxiety. Laying it down again stirs the "fount of feeling" still deeper. But now, as then, the matter has been carefully weighed before the responsibility is taken. Does the reader doubt it? Then listen to what is involved in the step: Personally, to the writer, is lost a great portion of his interest in the profession, and a much greater portion of the profession's interest in him. Persons who seldom meet and converse together, cease to be warmly interested in each other. The monthly conversations of the "Register" with its readers no longer form a tie between the writer and his brethren. Now they are as brethren of a scattered household, and he is to them as a stranger. This step involves a loss of interest in the scientific progress of the profession. The responsibility of position is a spur to effort. Take away the spur, and the pace of a jaded horse is slackened. This step, or at least the state of circumstances rendering it necessary, involves, too, a secondary, or subordinate position in the profession. True there may be no positive retrogression; but in the rapid advancement of professional science, he who fails to make progress is soon far in the rear. But the step must be taken, though all this, and far more, is felt to be true. More, far more than a "farewell" to the readers of the "Register" is here involved. A farewell to boyhood's hopes, and manhood's ambition! A farewell to an inordinate — unholy (?) desire to fall in the front rank of the advance corps of our professional army! Here is an invitation to join the benighted and the befogged in the profession—to follow where others lead—to leave the fresh flowers of science to others, and to crop the withered plants of professional byways. Here is a prospect of a popularity with the profession equal to that of the drone at killing time. Even now is a vision of formal friendship, where the transparent smile fails to hide the subjacent sentiment, "POOR OLD FOGY!" "I pray thee let me go over and see that good land, that goodly mountain and Lebanon;" but a view from Pisgah was all that was granted.

Well,—in resigning the pen, it is pleasant to remember that, in all but a single instance, good humor flowed from it as freely as the ink. For just half a page written, when petulant, the reader's pardon is asked. No apology is offered for other pages. They are as good as were promised. In retiring, the "Register"

is kindly commended to the good will of the profession. It is worthy of support, of the warmest encouragement. In keeping it alive the first two years of the war, the publisher manifested an energy hitherto unknown in our profession. His efforts should be appreciated.

And now, this step is taken without counsel from any one. No one could give advice intelligently. The reasons for it are not known to others. Hence it remains but to say, FAREWELL.

GEORGE WATT.

The above makes us exceedingly sad. We have had business connection with DR. WATT for nearly twenty years, and always with the utmost harmony, and now the last link is broken, not as we conceive because he, or any one wished it so, but because circumstances that he, or no one else could control, demands that it should be so. We hope that it will not always be thus. We will endeavor as well as we can to submit. We know there are hundreds in the profession who will greatly regret this withdrawal. T.

---

### "WHAT FORM OF MERCURY IS CONTAINED IN THE RUBBER AS A BASE FOR ARTIFICIAL TEETH?"

*Correspondent.*

We have frequently been asked this question, and others similar to it, and we will endeavor now to make a sort of general reply, or in other words answer them all at once:

The red coloring matter in the ordinary prepared rubber, for for the Dentists use, is a bi-sulphuret of mercury, which as ordinarily procured, is a brilliant, crystalline mass, of a deep red color, and fibrous texture.

It is inodorous and tasteless, and insoluble in water, alcohol, nitric, muriatic, or cold sulphuric acids, or by the solutions of the caustic alkalies, but is soluble in nitro-muriatic acid; thus we see it is in no condition to act injuriously when used in connection with rubber for the purpose specified. For, in the firse place, this coloring matter is so thoroughly incorporated with the vulcanized rubber, that it can not be liberated; and in the next place, there is no solvent for it in the mouth, even were it liberated; and in the next place, could it be disengaged from the surface of the rubber, and were there a solvent for it present, and the solution swallowed, it would not in one case in ten thousand, make any impression, for the ordinary dose, taken internally, is from ten grains to half a drachm.

So far as danger in this direction is concerned, we regard it very much less, than in the use of silver plates. T.

### NERVE EXTRACTORS.

A short time ago, we received some very fine nerve extractors, from Dr. F. Field, of Waltham, Mass. The form is that of a very fine four sided untempered steel broach, barbed on two corners. These broaches pass into a steel socket handle that accompanies them. They are so fine that they will pass into any nerve canal, and so perfectly barbed that they will scarcely ever fail to bring away the pulp, at least with ordinary skill.

We presume they may be obtained of either Dr. Field or of S. S. White.

### DELAY.

This No. of the REGISTER is very much delayed, and chiefly on account of our ill-health for the past month, from which we hope soon to be entirely recovered, so that our work may go on as usual. Our correspondence is also much behind, concerning which we hope our friends will have patience.

### STILL AT THE OLD STAND.

Dr. J. M. Brown, corner of Fourth and Walnut Streets, still continues his Dental furnishing business in all its departments. We believe the Dr. has the oldest establishment of the kind in the West. He has been a tried and faithful servant of the profession; he knows what the dentists want, and they know that he has the ability and the will to supply them. If others have succeeded, he has the consolation of knowing that he showed them the way.

### CLOSE OF THE VOLUME.

This number closes the Eighteeenth Volume of the REGISTER, and with it comes a very material, and much to be regretted change in the editorial management. In regard to it we have a very peculiar feeling of loneliness, and we hardly know what to say the REGISTER will be in the future; yet we propose to make it an acceptable journal to the profession, as much, or more so if possible, than hitherto. We shall continue the publication as heretofore, monthly, and of the same size; but we must rely largely upon the aid of our professional brethren, to sustain it with their pens, as well as with their money.

We do not propose to buy contributions, but will rely upon the interest which our brethren may have in sustaining a professional literature.

While we give hours of weary toil, almost daily, from the beginning to the end of the year, to this work, without one farthing of pecuniary renumeration, we deem it hardly just that we should be called upon to pay for a few hours labor, in the production of an article for our pages, while the writer ought to feel amply remunerated for having said some good things to the profession.

We shall continue to gather from every source, and present that which we conceive to be of the most interest to the profession.

We have a few delinquents upon our books, who we hope will be delinquents no longer, when they know how badly we need what they owe us. With this number we send out bills to all such.

